# Orthostatic Hypotension and Elevated Resting Heart Rate Predict Low-Energy Fractures in the Population: The Malmö Preventive Project

**DOI:** 10.1371/journal.pone.0154249

**Published:** 2016-04-28

**Authors:** Viktor Hamrefors, Maria Härstedt, Anna Holmberg, Cecilia Rogmark, Richard Sutton, Olle Melander, Artur Fedorowski

**Affiliations:** 1 Department of Clinical Sciences, Faculty of Medicine, Lund University, Malmö, Sweden; 2 Department of Medical Imaging and Physiology, Skåne University Hospital, Malmö, Sweden; 3 Department of Orthopaedics, Skåne University Hospital, Malmö, Sweden; 4 National Heart and Lung Institute, Imperial College, St Mary’s Hospital Campus, London, UK; 5 Department of Internal Medicine, Skåne University Hospital, Malmö, Sweden; 6 Department of Cardiology, Skåne University Hospital, Malmö, Sweden; The University of Tokyo, JAPAN

## Abstract

**Background:**

Autonomic disorders of the cardiovascular system, such as orthostatic hypotension and elevated resting heart rate, predict mortality and cardiovascular events in the population. Low-energy-fractures constitute a substantial clinical problem that may represent an additional risk related to such autonomic dysfunction.

**Aims:**

To test the association between orthostatic hypotension, resting heart rate and incidence of low-energy-fractures in the general population.

**Methods and Results:**

Using multivariable-adjusted Cox regression models we investigated the association between orthostatic blood pressure response, resting heart rate and first incident low-energy-fracture in a population-based, middle-aged cohort of 33 000 individuals over 25 years follow-up.

The median follow-up time from baseline to first incident fracture among the subjects that experienced a low energy fracture was 15.0 years. A 10 mmHg orthostatic decrease in systolic blood pressure at baseline was associated with 5% increased risk of low-energy-fractures (95% confidence interval 1.01–1.10) during follow-up, whereas the resting heart rate predicted low-energy-fractures with an effect size of 8% increased risk per 10 beats-per-minute (1.05–1.12), independently of the orthostatic response. Subjects with a resting heart rate exceeding 68 beats-per-minute had 18% (1.10–1.26) increased risk of low-energy-fractures during follow-up compared with subjects with a resting heart rate below 68 beats-per-minute. When combining the orthostatic response and resting heart rate, there was a 30% risk increase (1.08–1.57) of low-energy-fractures between the extremes, i.e. between subjects in the fourth compared with the first quartiles of both resting heart rate and systolic blood pressure-decrease.

**Conclusion:**

Orthostatic blood pressure decline and elevated resting heart rate independently predict low-energy fractures in a middle-aged population. These two measures of subclinical cardiovascular dysautonomia may herald increased risks many years in advance, even if symptoms may not be detectable. Although the effect sizes are moderate, the easily accessible clinical parameters of orthostatic blood pressure response and resting heart rate deserve consideration as new risk predictors to yield more accurate decisions on primary prevention of low-energy fractures.

## Introduction

Changes in autonomic control of the cardiovascular system may adversely affect various aspects of health [1]. Orthostatic hypotension (OH) is a common manifestation of autonomic dysfunction [2, 3] that occasionally leads to cerebral hypoperfusion and syncope, either directly by the decrease in blood pressure (BP) or indirectly by triggering the vasovagal reflex, but is often asymptomatic [4]. OH conveys independent prognostic information concerning mortality [5] with an underlying increased risk of death due to cardiovascular disease (CVD), injuries, neurodegenerative, and respiratory diseases [6, 7]. In parallel, an elevated resting heart rate (RHR), which correlates with OH [8], has been linked with increased general and cardiovascular mortality [9, 10] independently of traditional risk factors.

Hemodynamic impairment during orthostasis is a well-known risk factor for traumatic falls [6, 11, 12]. Low-energy fractures, resulting from low-energy traumas such as a fall from standing position, constitute a substantial clinical problem [13, 14]. Whereas several risk factors for low-energy fractures have been identified in the population [15, 16], the specific underlying hemodynamic factors have not been thoroughly studied. Importantly, in pharmacological primary prevention of low-energy fractures with e.g. bisphosphonates, there is a clinical need to sharpen risk prediction to target subjects at highest risk and thus reduce the number needed to treat [17]. Indeed, non-pharmacological primary prevention has a similar demand to focus interventions on the appropriate individuals. Also, in general terms, low-energy fractures can plausibly be regarded as a surrogate marker of falls, thus being a suitable endpoint for the assessment of risks related to autonomic dysfunction.

In this study, we investigated longitudinal association of orthostatic BP fall, RHR and their combination at baseline with incident low-energy-fractures in a middle-aged cohort of 33 000 individuals.

## Methods

### Study population

The Malmö Preventive Project (MPP) is a population-based prospective cohort study in the city of Malmö, in southern Sweden [18]. The primary aim was to screen for CVD among large strata of the adult population. Subjects from birth cohorts in Malmö were invited by mail and a total of 33346 (22444 men and 10902 women, mean age 45 years; range 26–61 years) inhabitants of Malmö, born between 1921 and 1948 attended the screening program and were examined between 1974 and 1992. The overall participation among invited subjects was 71% [19].

The current analysis is a retrospective review of the prospective study of MPP. The present study included subjects from MPP with complete data on age, sex, body-mass index (BMI) and follow-up data of low-energy fractures (see below), rendering a total of 33139 subjects eligible for analysis.

### Baseline examination

Subjects were asked to abstain from food, alcohol and tobacco for 12 h prior to the baseline examination, which was performed by trained nurses in the morning. BP was measured using the auscultatory method with a mercury sphygmomanometer on the right arm at heart level. BP was measured twice in the supine position and twice after one minute of standing. The mean value of two readings was recorded for each position and rounded to nearest 5 mmHg. The heart rate was measured twice by palpation over one minute in supine position. After the examination, the participants were asked to fill a questionnaire focused on personal and family history of CVD, hypertension, diabetes, cancer, smoking habits, and lifestyle pattern.

A detailed description of recruitment and screening procedures of MPP may be found elsewhere [18, 20]. The MPP was approved by the Health Department of Malmö city (1972) and the retrospective analysis of the cohort was approved by IRB in Lund. All participants gave written informed consent. The data were anonymized before the analyses.

### Definition of the clinical variables at baseline used in the current study

Decrease in SBP and DBP, the components of orthostatic hemodynamic response, were assessed using a continuous/semi quantitative scale. Orthostatic SBP and DBP response (ΔSBP and ΔDBP) were defined as standing SBP/DBP minus supine SBP/DBP. The RHR was the mean of two measurements in supine position. BMI was calculated as weight in kilograms divided by the square of the height in meters. Antihypertensive treatment (AHT) was defined as a positive answer to the following question: Do you take medication for high blood pressure? Those who confirmed regular or occasional current smoking were classified as smokers. Diabetes was defined as fasting plasma glucose of ≥ 7.0 mmol/l, current pharmacological treatment of diabetes or self-reported history of diabetes. Previous myocardial infarction (MI) at baseline was recorded from the national Swedish patient register (which contains data from 1964- with complete coverage from 1987-).

### Follow-up and retrieval of end-points

Fracture data were obtained by linking the unique Swedish ten-digit personal identification number of included subjects with the register at the Department of Radiology at Malmö University Hospital as previously described [16]. In the city of Malmö, all emergency radiographic examinations are performed at the Department of Radiology of Skåne University Hospital and reports and films of fractures are stored. Fractures identified were confirmed through manual search of medical and radiological files. Previous studies have shown that at least 97% of all fractures experienced by citizens of Malmö can be identified this way [21]. Follow-up extended to December 31 2006.

Fractures were classified as high- or low-energy trauma depending on the cause of fracture, based on information in the radiographic reports. Fractures caused by falling from standing position or less energy were classified as low-energy fractures. Of all fractures, 97.7% had descriptions with adequate information about the degree of trauma. The fractures with insufficient information were classified as low-energy fractures based on the experience that it is highly unlikely that information about high-energy accidents is not reported. Fractures caused by high-energy trauma were excluded from all analyses, as were also pathological fractures caused by cancer or other bone diseases.

### Statistics

The hemodynamic parameters recorded at baseline (supine SBP/DBP, ΔSBP/DBP, and RHR) were related to first incident low-energy fracture using multivariable-adjusted Cox-regression models. We tested models entering age, sex and BMI as covariates (Model 1) as well as more comprehensive models including age, sex, BMI, AHT, diabetes, smoking, previous MI, and all measured hemodynamic parameters (supine SBP/DBP, ΔSBP/DBP and RHR) from the baseline examination (Model 2).

In order to evaluate the combined effect of RHR and OH on the risk of incident low energy fractures we constructed a combined RHR-OH-score for each individual subject. The score was constructed as follows: the study population was split into quartiles according to baseline RHR and ΔSBP, respectively. Thus, each individual was given a quartile number (1 for lowest, 4 for highest) for RHR and ΔSBP. The RHR-OH-score for each individual (range 2–8) was then constructed by summing the individual quartile number for RHR and ΔSBP, respectively. Additionally, in order to further investigate the specific contribution of RHR and ΔSBP and of their combined effect to the risk of incident low-energy-fractures, the 16 specific quartile combinations of RHR and ΔSBP that could be combined for the study subjects (i.e. subjects in [Q1 for RHR–Q2 ΔSBP], [Q1 for RHR—Q3 ΔSBP]…up to [Q4 for RHR- Q4 - ΔSBP] was tested in Cox-regression models in relation to the reference [Q1 for RHR–Q1 for ΔSBP].

**Fig 1:**
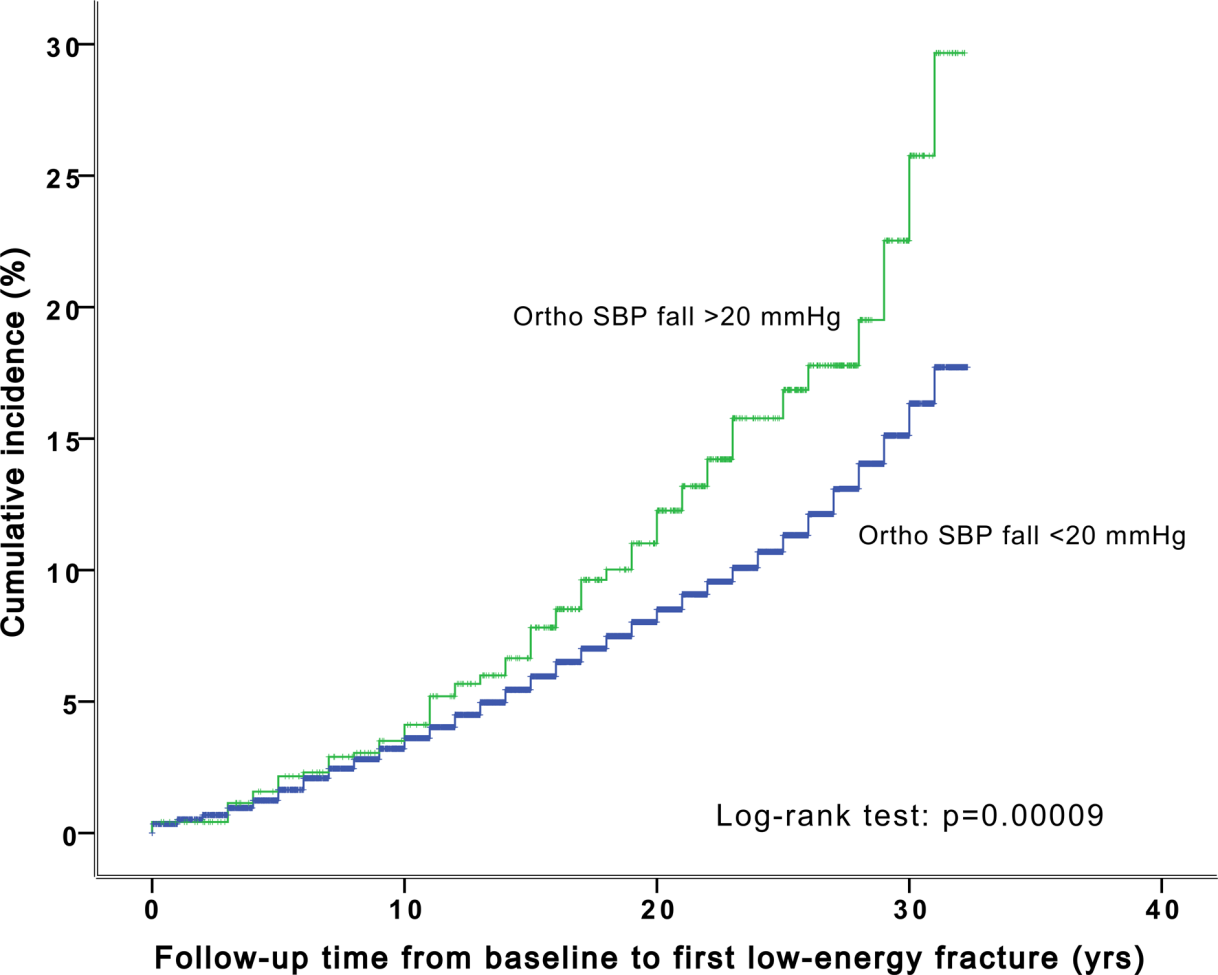
The cumulative incidence of low-energy fractures stratified by orthostatic decrease in systolic blood pressure over 20 mmHg in the Malmö Preventive Project population (n = 33 000).

**Fig 2:**
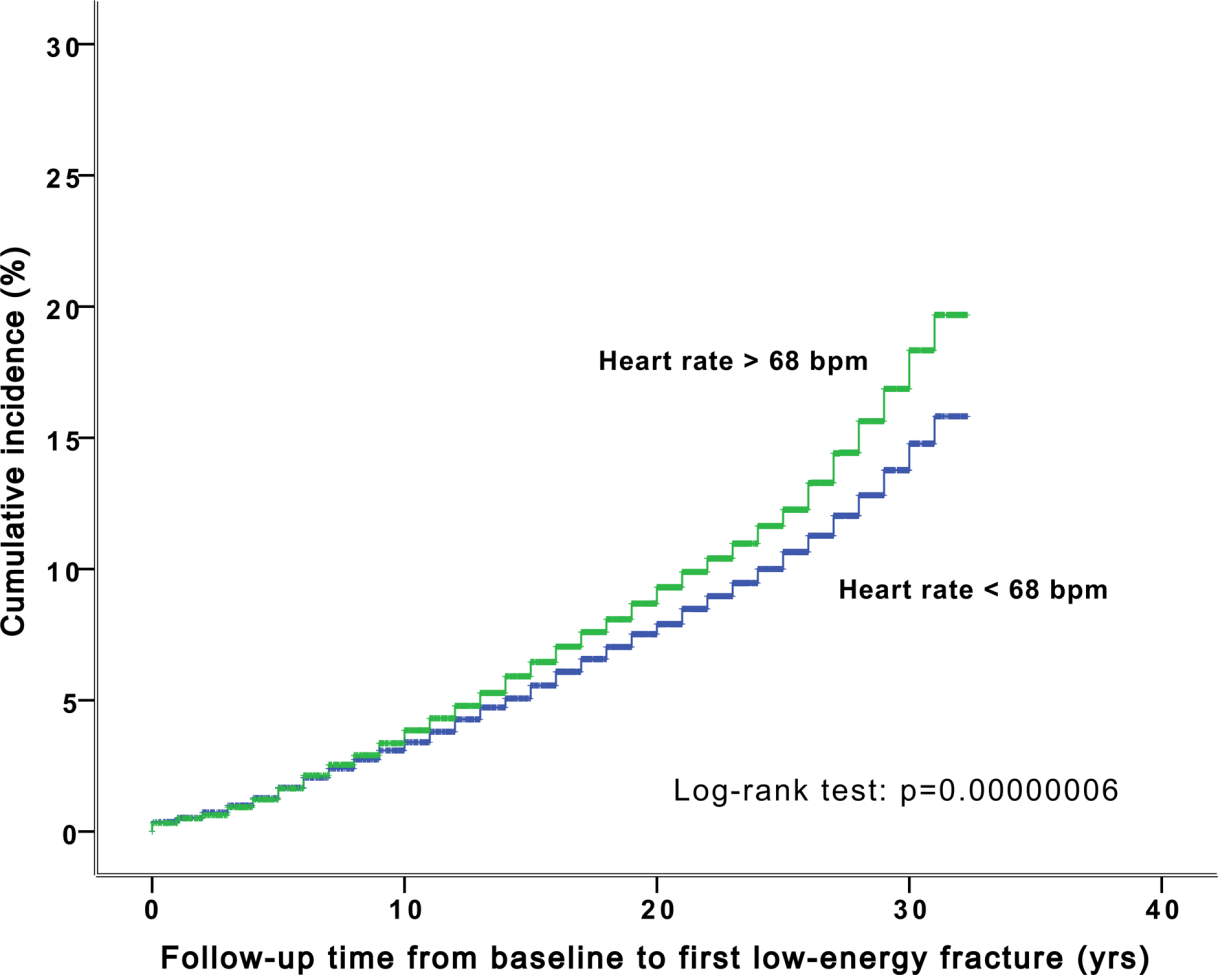
The cumulative incidence of low-energy fractures stratified by resting heart rate over 68 bpm in the Malmö Preventive Project population (n = 33 000).

The proportional-hazards assumption was confirmed by visual inspection of survival curves (Figs 1 and 2).

## Results

### Population characteristics

Baseline characteristics of the study population are shown in Table 1. There were 4.9 first incident low-energy fractures per 1000 person-years during a median follow-up time of 25.1 years in the whole population. The median follow-up time from baseline to first incident fracture among the subjects that experienced a low energy fracture was 15.0 years. The mean age at first low-energy fracture among these subjects was 63.5 years (10.7 years)

**Table 1:** Baseline characteristics.

Baseline characteristics of the study population (N = 33139)	
Age, years	45.6 (7.4)
Sex, % male	67.6
BMI, kg/m2	24.6 (3.6)
Current smoker, %	44.6
Supine SBP, mmHg	129.2 (15.6)
Supine DBP, mmHg	85.1 (9.5)
Postural SBP response, mmHg	-1.8 (7.4)
Postural DBP response, mmHg	2.3 (4.5)
Postural SBP decrease ≥ 20 mmHg, %	2.2
Orthostatic hypotension (postural decrease ≥ 20/10 mmHg), %	2.8
Resting heart rate, BPM	69 (10)
Antihypertensive treatment, %	5.4
Diabetes, %	4.7
Previous myocardial infarction, %	0.4

Values displayed as mean (SD) if not otherwise indicated. BMI = body mass index; SBP = systolic blood pressure; DBP = diastolic blood pressure; BPM = beats per minute.

### Relation between orthostatic blood pressure change and low energy fractures

The mean orthostatic change in SBP predicted incident low-energy fractures, in both models. The effect size was approximately 5% increased risk per 10-mmHg decrease in SBP in the fully adjusted model (Table 2). The relative risk increase could be observed for SBP fall in the range of 0–5 mmHg (3^rd^ quartile in the population, +12%), and it was even higher for those who demonstrated SBP fall >5 mmHg (4^th^ quartile, + 17%) (S1 Table). In contrast, the orthostatic DBP-response did not predict low energy fractures.

**Table 2:** Relation between hemodynamic parameters and first incident low energy fracture.

	Number of subjects (events)	HR	95% CI	*P* value
**Supine SBP**				
Model 1	32672 (3597)	1.002 per mmHg	1.000–1.004	0.128
Model 2*	32672 (3597)	1.002 per mmHg	1.000–1.004	0.066
**Supine DBP**				
Model 1	32667 (3596)	1.001 per mmHg	0.997–1.004	0.691
Model 2*	32667 (3596)	1.002 per mmHg	0.998–1.005	0.390
**ΔSBP**				
Model 1	32610 (3584)	1.006 per - ΔmmHg	1.002–1.011	0.005
Model 2**	32610 (3584)	1.005 per - ΔmmHg	1.001–1.010	0.022
**ΔDBP**				
Model 1	32594 (3582)	1.007 per - ΔmmHg	1.000–1.015	0.052
Model 2***	32594 (3582)	1.006 per - ΔmmHg	0.999–1.014	0.109
**RHR**				
Model 1	32530 (3573)	1.009 per BPM	1.005–1.012	<0.001
Model 2****	32461 (3559)	1.008 per BPM	1.005–1.012	<0.001

Model 1: Includes covariates age, sex, BMI. Model 2

* Includes covariates age, sex, BMI, AHT, smoking, diabetes, previous MI

** Includes covariates age, sex, BMI, AHT, smoking, diabetes, previous MI, SBP supine

***’ Includes covariates age, sex, BMI, AHT, smoking, diabetes, previous MI, DBP supine

****’ Includes covariates age, sex, BMI, AHT, diabetes, smoking, previous MI, SBP supine, ΔSBP in standing. HR = hazard ratio; 95% CI = 95% Confidence interval; SBP = systolic blood pressure; DBP = diastolic blood pressure; RHR = resting heart rate.

When analyzing the type of fractures predicted by a decrease in SBP we found (in the fully adjusted models) an association with vertebral fragility fractures (221 fractures; 24 percent risk increase per—10 mmHg; *P* = 0.008) whereas there were no significant associations with radius, skull and hip fractures (S2 Table).

### Relation between RHR and low- energy fractures

RHR predicted incident low-energy fractures in both models, the latter model also including supine SBP and orthostatic change in SBP as covariates. The effect size was approximately 8% increased risk per 10 beats per minute (bpm) in the fully adjusted model (Table 2). Subjects with a RHR exceeding 68 bpm (= median RHR) had 18% (95% CI 1.10–1.26; *P* < 0.001) increased risk of low-energy fractures during follow-up compared with subjects having a RHR below 68 bpm.

There was a linear association between RHR and number of low energy fractures in individual subjects (P = 0.011) and the odds ratio (OR) of suffering more than one fracture (compared with one or zero) was 1.10 (95% CI 1.03–1.16; P = 0.002) per RHR-quartile in a logistic regression model, adjusted as in model 2.

On analysis of specific fractures, we found (in the fully adjusted models) an association with distal radius fractures (1020 fractures; 14% risk increase per 10 bpm; *P* <0.001) and a borderline-significant association with vertebral fragility fractures (219 fractures; 14% risk increase per 10 bpm; *P* = 0.050), whereas there were no associations with hip fractures and skull fractures (S2 Table).

### The combined value of OH and RHR on predicting low-energy fractures

**Fig 3:**
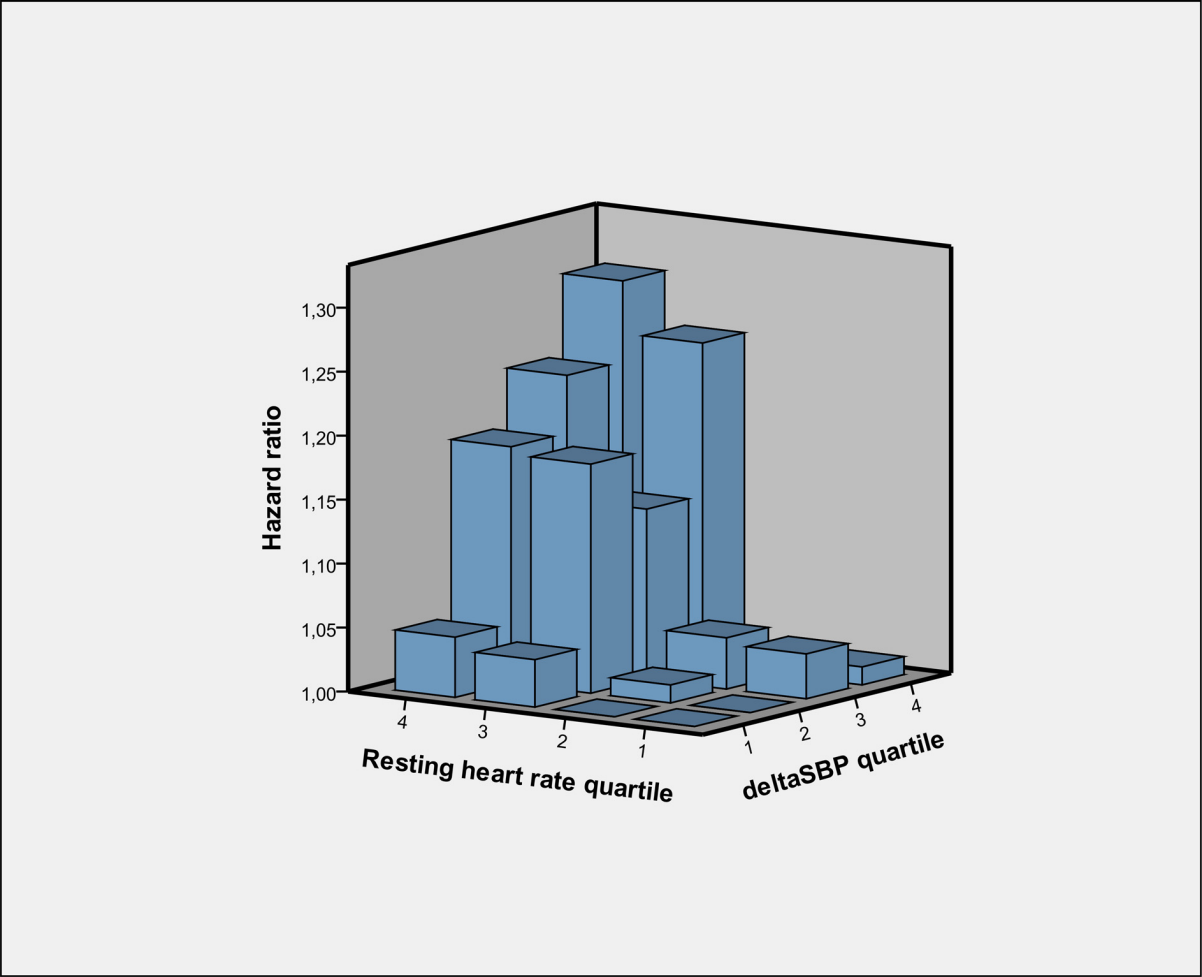
Quartile specific hazard ratios concerning resting heart rate and orthostatic change in systolic blood pressure (SBP) in relation to first incident low-energy fracture. Hazard ratio = 1.00 (reference) for quartile 1/1 of resting heart rate/ΔSBP. Model includes covariates age, sex, body-mass-index, antihypertensive treatment, smoking, diabetes, previous myocardial infarction, SBP in supine. Results are detailed in S3 Table.

When combining orthostatic SBP-decrease and RHR there was a 30% risk increase of first incident low energy fracture (95% CI 1.08–1.57; *P* <0.001) between the extremes of the RHR-OH-score (i.e. subjects in the fourth compared with the first quartiles of both RHR and ΔSBP). When stratifying subjects by quartiles of both RHR and ΔSBP in relation to first incident low energy fracture the effect of orthostatic SBP-decrease on risk of low energy fractures was evident predominantly in the upper quartiles of RHR (Fig 3, S3 Table).

## Discussion

In this study, we evaluated two hemodynamic parameters, orthostatic blood pressure-fall and resting heart rate, in relation to first incident low-energy fracture during a median follow-up of 25 years in a population-based middle-aged cohort of 33 000 subjects. The orthostatic systolic blood pressure-decrease at baseline predicted low-energy fractures, also after adjusting for covariates known to be associated with fragility fractures such as smoking and antihypertensive therapy [15, 16]. This is in line with our previous observation of a strong association between impaired orthostatic BP regulation and fatal injuries in the same population [6]. As can be seen the S1 Table, the relative risk increase could be observed for SBP fall in the range of 0–5 mmHg and it was higher for those who demonstrated SBP fall >5 mmHg. Thus, although the current guidelines recommend SBP decline > 20mmHg on standing as the diagnostic criteria for OH, any drop in BP during orthostatic challenge indicate an increased risk for low-energy fracture. It is noteworthy that OH is often asymptomatic, and the first recognized symptom may be a complete loss of consciousness resulting in trauma [22]. Apart from this finding, we also determined a strong association between elevated RHR and low-energy fractures, independently of supine SBP and orthostatic SBP-decrease. Finally, there was a strong association with risk of first incident low-energy fracture when combining RHR and orthostatic BP-fall in a model, observing a 30% risk increase between subjects at the extremes of the combination of both variables.

In a recent meta-analysis, Ricci and colleagues reported that OH is associated with increased risk of death and CVD [5] regardless of studied population. Further, higher RHR has been reported to predict mortality and cardiovascular events [9, 23] in the general population. Our study is, to the best of our knowledge, the first to show a prospective association between OH, RHR and incident low-energy-fractures among middle-aged adults over a long follow-up.

It is likely that many low-energy-fractures are caused by falls and that these falls may in turn be provoked by a temporary cerebral hypoperfusion resulting from impaired BP response to orthostasis. As such, low-energy fractures may serve as a surrogate variable for falls in the population. Supporting this hypothesis, we found a stronger effect of both OH and RHR on fracture types traditionally associated with falls, such as distal radius and vertebral fragility fractures (even though the latter may also occasionally be non-traumatic).

The main potential clinical implication of our results is the possibility that OH and RHR may be used to sharpen clinical risk prediction of low-energy fractures. Low energy fractures constitute a huge clinical problem [13, 14], often being a complicating factor in patients with multiple comorbidities treated in the internal medicine ward. A number of risk factors for low-energy fractures have been identified [15, 16], however, in the primary pharmacological prevention of low-energy fractures with e.g. bisphosphonates, there is still a clinical need to improve risk prediction in order to better target subjects at highest risk and thus reduce the number needed to treat [17]. Non-pharmacological primary prevention has a similar demand to focus interventions on the right individuals. We were unable to adjust for measures of bone density; however, we find it unlikely that bone density would be a real confounder as it is likely to be related only to the outcome and not to the exposures (OH and RHR). Accordingly, even though the effect sizes are moderate, we suggest that OH and RHR may be used as potential additional predictors in clinical decisions on fracture preventive therapy.

As for mechanistic aspects of the current results, we propose some explanations why OH and particularly RHR are independently associated with low-energy injuries. The first and most obvious is that these variables are merely markers of poor physical fitness or an impaired health status, which in turn would be correlated with traditional risk factors for fragility fractures such as diabetes or smoking [15, 16]. However, the association between RHR and mortality has been found to hold true irrespective of physical fitness [9] meaning that RHR is likely to convey an independent prognostic value. As expected, RHR correlated strongly with a number of variables in our study population (S5 Table). However, the effect size of RHR on predicting low-energy fractures was not reduced when orthostatic SBP-response or other factors supposedly associated with impaired health were included in the model. Furthermore, the strong predictive value of RHR on incident low-energy fractures persisted when analyses were done separately in untreated hypertensives as well as in normotensives (S4 Table). This supports the hypothesis that RHR is not only a mere marker of impaired health status. Accordingly, we propose that a higher RHR may be a marker of subtle autonomic dysfunction associated with sympathetic hyper-activation, an explanation in parallel with that between elevated RHR and CVD [9, 23]. Advancing age, diabetes, hypertension—other CVD risk factors that are all associated with OH—share the potential to impair or override autonomic mechanisms [3] resulting in a higher RHR. Thus, subclinical forms of the above conditions may independently and additively influence RHR. Also, elevated heart rate may be an expression of impaired baroreflex function, involved in the pathogenesis of OH [24], and characterized by enhanced sympathetic activity and withdrawal of parasympathetic control. Regardless of whether subclinical CVD or a malfunctioning baroreflex is the cause, a higher RHR is likely to indicate the presence of autonomic dysfunction. We hypothesize that when this initially subtle autonomic dysfunction evolves to a manifest condition, it may eventually lead to overt OH or possibly arrhythmias causing falls and fall-related injuries.

Alternative mechanisms that might link elevated RHR with fall-related injuries include the possibility of an undiagnosed state that increases the risk of pre-syncope and syncope such as postural orthostatic tachycardia syndrome (POTS) [25]. Also, a higher heart rate implies that the cardiovascular system is already challenged at rest. Thus, subjects with a higher heart rate may be prone to cerebral hypoperfusion during orthostatic challenge, due to reduced circulatory reserve. The elevated heart rate at rest and limited capacity for tolerating orthostatic changes may partly be determined by genetic factors associated with cardiac and neuroendocrine conditions [26, 27].

Regarding the long time span between baseline screening and the index event, a similar temporal delay was observed in MPP in relation to all-cause mortality, and cardiovascular events such as myocardial infarction [8]. Thus, the signs of subclinical cardiovascular dysautonomia may herald increased risks many years in advance, even if the symptoms may not be detectable. This might be due to compensatory mechanisms such as cerebral autoregulation, protecting the brain tissue against systemic hemodynamic fluctuations [28]. Consequently, effects of cardiovascular dysautonomia may not be directly perceived by an affected individual but ageing may eventually provoke failure of compensatory mechanisms and unexpected fall leading to trauma.

Although asymptomatic to a large extent, OH is a clinically well recognized problem, especially in the elderly in whom the consequences of OH (such as a fracture) may frequently be the first manifestation [4]. Various drugs have been tested as symptomatic relief of OH, however, without convincing data and international consensus on their efficacy [5]. Concerning heart rate regulation, beta-blockers have a role in the primary and secondary prevention and treatment of most types of CVD [29] and are sometimes used as symptomatic treatment in inappropriate sinus tachycardia (IST) and POTS, as is the heart rate modulating drug Ivabradine [25]. Whether or not heart rate modulating drugs may have a role in treating subtle autonomic dysfunction remains to be tested. Regardless of these considerations, OH and RHR may, as previously discussed, possibly enhance decision making of fracture-preventive therapy in order to reduce a consequence of such subtle autonomic dysfunction.

The main strengths of this study are the large number of subjects, long follow-up and access to reliable case data. The major limitation of this study is the lack of data on heart rate and 3-min BP on standing which were not recorded in MPP. Naturally, the current guidelines on OH [2] were not available at the time of the baseline examination of MPP. Another limitation is that we assumed that most cases of low-energy-fractures were indeed caused by a fall provoked by reduced cerebral circulation during a maladaptive BP response. However, some low-energy-fractures are likely to occur in other settings. Finally, for the whole MPP cohort orthostatic response was examined only at baseline, indicating that we have been unable to evaluate how prospective changes in hemodynamic parameters were related to outcome.

In conclusion, we have shown that orthostatic blood pressure decline, elevated resting heart rate and their combination are strong independent predictors of low-energy fractures in a middle-aged population and as such, potential signs of subtle autonomic dysfunction in these subjects. Orthostatic blood pressure response and resting heart rate deserve consideration as new tools for risk prediction in order to obtain more accurate clinical decisions on non-pharmacological and pharmacological primary prevention of low-energy fractures.

## Supporting Information

S1 Table Quartile-specific estimates of systolic orthostatic blood pressure response. (DOCX)

S2 Table Relation between hemodynamic parameters and first incident low-energy-fracture subtypes. (DOCX)

S3 Table Quartile-specific relation between resting heart rate, orthostatic systolic blood pressure change and risk of first incident low-energy fracture. (DOCX)

S4 Table **Relation between hemodynamic parameters and first incident low energy fractures in untreated hypertensives (A) and in normotensives (B).**
(DOCX)

S5 Table Correlation between resting heart rate and other variables in the study population. (DOCX)
