# Supplementary material for: Orthostatic Hypotension and Elevated Resting Heart Rate Predict Low-Energy Fractures in the Population: The Malmö Preventive Project
Source: PLoS One. 2016 Apr 28;11(4):e0154249. doi: 10.1371/journal.pone.0154249 (PMC4849675; doi:10.1371/journal.pone.0154249)
Supplement: S1 Table — (DOCX) [file pone.0154249.s001.docx]

**S1 Table.** **Quartile-specific estimates of systolic orthostatic blood pressure response.**

| **ΔSBP divided in quartiles in the study population** | | | | | | |
| --- | --- | --- | --- | --- | --- | --- |
| **Quartile** | **ΔSBP mean** |  | **SD** |  |  | **Median (minimum; maximum)** |
| Q1 | 7.7 |  | 3.6 |  |  | 7,5 (3.0; 47.5) |
| Q2 | 1.0 |  | 1.2 |  |  | 0.0 (0.0; 2.5) |
| Q3 | -3.7 |  | 1.3 |  |  | 4.0 (-5.0; -0.5) |
| Q4 | -11.7 |  | 5.2 |  |  | -10.0 (-70.0; -5.5) |

| **Quartiles of - ΔSBP as a predictor of incident low-energy fractures** | | | | | | |
| --- | --- | --- | --- | --- | --- | --- |
|  |  |  | **HR** | **95 % CI** | **P-value** |  |
| - ΔSBP Q1 |  |  | 1.00 |  |  |  |
| - ΔSBP Q2 |  |  | 1.09 | 0.98-1.20 | 0.100 |  |
| - ΔSBP Q3 |  |  | 1.12 | 1.01-1.23 | 0.029 |  |
| - ΔSBP Q4 |  |  | 1.17 | 1.05-1.29 | 0.003 |  |
