# Supplementary material for: Orthostatic Hypotension and Elevated Resting Heart Rate Predict Low-Energy Fractures in the Population: The Malmö Preventive Project
Source: PLoS One. 2016 Apr 28;11(4):e0154249. doi: 10.1371/journal.pone.0154249 (PMC4849675; doi:10.1371/journal.pone.0154249)
Supplement: S2 Table — (DOCX) [file pone.0154249.s002.docx]

**S2 Table.** **Relation between hemodynamic parameters and first incident low-energy-fracture subtypes.**

|  | **Number of subjcets (events)** | **HR** | **95 % CI** | ***P* value** |
| --- | --- | --- | --- | --- |
| **Radius**  ΔSBP  ΔDBP  RHR | 32610 (1031) 32594 (1031) 32461 (1020) | 1.003 per - ΔmmHg  0.999 per - ΔmmHg 1.014 per BPM | 0.995-1.011 0.985-1.013 1.007-1.020 | 0.458 0.889 <0.001 |
| **Vertrebral fragility**  ΔSBP  ΔDBP  RHR | 32610 (221)  32594 (221)  32461 (219) | 1.024 per - ΔmmHg  1.033 per - ΔmmHg  1.014 per BPM | 1.006-1.042  1.002-1.064  1.000-1.028 | 0.008  0.035  0.050 |
| **Skull**  ΔSBP  ΔDBP  RHR | 32610 (43)  32594 (43)  32461 (43) | 1.036 per - ΔmmHg  1.015 per - ΔmmHg  1.023 per BPM | 0.994-1.080  0.947-1.088  0.993-1.053 | 0.097  0.676  0.132 |
| **Hip**  ΔSBP  ΔDBP  RHR | 32610 (445)  32594 (443)  32461 (443) | 1.006 per - ΔmmHg  1.015 per - ΔmmHg  1.009 per BPM | 0.993-1.019  0.994-1.370  0.999-1.019 | 0.334  0.157  0.088 |
| The models used are the same as shown for Model 2 in Table 2  SBP = systolic blood pressure; DBP = diastolic blood pressure; RHR = resting heart rate. | | | | |
|  |  |  |  |  |
|  |  |  |  |  |
