# Supplementary material for: Orthostatic Hypotension and Elevated Resting Heart Rate Predict Low-Energy Fractures in the Population: The Malmö Preventive Project
Source: PLoS One. 2016 Apr 28;11(4):e0154249. doi: 10.1371/journal.pone.0154249 (PMC4849675; doi:10.1371/journal.pone.0154249)
Supplement: S3 Table — (DOCX) [file pone.0154249.s003.docx]

**S3 Table.** **Quartile-specific relation between resting heart rate, orthostatic systolic blood pressure change and risk of first incident low-energy fracture.**

| RHR Quartile | - ΔSBP Quartile | Hazard ratio | 95 % CI | *P* value |
| --- | --- | --- | --- | --- |
| 1 | 1 | 1.00 (ref) |  |  |
| 1 | 2 | 0.95 | 0.78-1.15 | 0.606 |
| 1 | 3 | 1.04 | 0.85-1.26 | 0.732 |
| 1 | 4 | 1.01 | 0.82-1.25 | 0.896 |
| 2 | 1 | 0.89 | 0.72-1.11 | 0.305 |
| 2 | 2 | 1.01 | 0.84-1.23 | 0.888 |
| 2 | 3 | 1.04 | 0.86-1.27 | 0.693 |
| 2 | 4 | 1.01 | 0.82-1.24 | 0.926 |
| 3 | 1 | 1.04 | 0.83-1.29 | 0.746 |
| 3 | 2 | 1.18 | 0.97-1.43 | 0.095 |
| 3 | 3 | 1.13 | 0.93-1.38 | 0.216 |
| 3 | 4 | **1.25** | **1.03-1.53** | **0.026** |
| 4 | 1 | 1.05 | 0.84-1.30 | 0.679 |
| 4 | 2 | 1.19 | 0.97-1.44 | 0.089 |
| 4 | 3 | **1.23** | **1.01-1.49** | **0.036** |
| 4 | 4 | **1.29** | **1.07-1.57** | **0.009** |
| Cox regression model includes covariates age, sex, BMI, AHT, smoking, diabetes, previous MI, SBP supine. RHR = resting heart rate. ΔSBP = decrease in systolic blood pressure on standing. | | | | |
