# Supplementary material for: Orthostatic Hypotension and Elevated Resting Heart Rate Predict Low-Energy Fractures in the Population: The Malmö Preventive Project
Source: PLoS One. 2016 Apr 28;11(4):e0154249. doi: 10.1371/journal.pone.0154249 (PMC4849675; doi:10.1371/journal.pone.0154249)
Supplement: S4 Table — Relation between hemodynamic parameters and first incident low energy fractures in untreated hypertensives (A) and in normotensives (B). (DOCX) [file pone.0154249.s004.docx]

**S4A Table. Relation between hemodynamic parameters and first incident low energy fractures in untreated hypertensives (N = 11462).**

|  | **Number of subjects (events)** | **HR** | **95 % CI** | ***P* value** |
| --- | --- | --- | --- | --- |
| **ΔSBP** |  |  |  |  |
| Model 1 | 11371 (1397) | 1.008 per - ΔmmHg | 1.001 - 1.014 | 0.024 |
| Model 2* | 11371 (1397) | 1.006 per - ΔmmHg | 0.999 - 1.013 | 0.088 |
| **ΔDBP** |  |  |  |  |
| Model 1 | 11366 (1395) | 1.013 per - ΔmmHg | 1.001-1.025 | 0.039 |
| Model 2** | 11366 (1395) | 1.011 per - ΔmmHg | 0.999-1.023 | 0.080 |
| **RHR** |  |  |  |  |
| Model 1 | 11325 (1391) | 1.008 per BPM | 1.004-1.013 | 0.001 |
| Model 2*** | 11301 (1387) | 1.007 per BPM | 1.001-1.012 | 0.012 |
|  |  |  |  |  |
| Model 1: Includes covariates age, sex, BMI.  Model 2:  * Includes covariates age, sex, BMI, smoking, diabetes, previous MI, SBP supine,  ** Includes covariates age, sex, BMI, smoking, diabetes, previous MI, DBP supine,  ***’ Includes covariates age, sex, BMI, diabetes, smoking, previous MI, SBP supine, ΔSBP in standing  HR = hazard ratio; 95 % CI = 95 % Confidence interval; SBP = systolic blood pressure; DBP = diastolic blood pressure; RHR = resting heart rate. | | | | |

**S4B Table. Relation between hemodynamic parameters and first incident low energy fractures in normotensives (N = 19816).**

|  | **Number of subjects (events)** | **HR** | **95 % CI** | ***P* value** |
| --- | --- | --- | --- | --- |
| **ΔSBP** |  |  |  |  |
| Model 1 | 19456 (1963) | 1.004 per - ΔmmHg | 0.998 - 1.011 | 0.213 |
| Model 2* | 19456 (1963) | 1.005 per - ΔmmHg | 0.998 - 1.012 | 0.133 |
| **ΔDBP** |  |  |  |  |
| Model 1 | 19448 (1963) | 1.005 per - ΔmmHg | 0.995-1.015 | 0.328 |
| Model 2** | 19448 (1963) | 1.007 per - ΔmmHg | 0.997-1.017 | 0.195 |
| **RHR** |  |  |  |  |
| Model 1 | 19421 (1958) | 1.008 per BPM | 1.003-1.013 | 0.001 |
| Model 2*** | 19385 (1950) | 1.010 per BPM | 1.004-1.015 | <0.001 |
|  |  |  |  |  |
| Model 1: Includes covariates age, sex, BMI.  Model 2:  * Includes covariates age, sex, BMI, smoking, diabetes, previous MI, SBP supine,  ** Includes covariates age, sex, BMI, smoking, diabetes, previous MI, DBP supine,  ***’ Includes covariates age, sex, BMI, diabetes, smoking, previous MI, SBP supine, ΔSBP in standing  HR = hazard ratio; 95 % CI = 95 % Confidence interval; SBP = systolic blood pressure; DBP = diastolic blood pressure; RHR = resting heart rate. | | | | |
