# Supplementary material for: Orthostatic Hypotension and Elevated Resting Heart Rate Predict Low-Energy Fractures in the Population: The Malmö Preventive Project
Source: PLoS One. 2016 Apr 28;11(4):e0154249. doi: 10.1371/journal.pone.0154249 (PMC4849675; doi:10.1371/journal.pone.0154249)
Supplement: S5 Table — (DOCX) [file pone.0154249.s005.docx]

**S5 Table. Correlation between resting heart rate and other variables in the study population.**

|  | **Pearson correlation coefficient** | **P-value** |
| --- | --- | --- |
| **Age** | 0.038 | <0.001 |
| **BMI** | 0.073 | <0.001 |
| **SBP supine** | 0.307 | <0.001 |
| **DBP supine** | 0.244 | <0.001 |
| **- ΔSBP** | 0.084 | <0.001 |
| **- Δ DBP** | -0.007 | 0.215 |

|  | **Resting heart rate (SD)** | **P-value for difference (Student’s T-test)** |
| --- | --- | --- |
| **Sex**  Male  Female | 68.8 BPM (10)  70.0 (9) | <0.001 |
| **Smoking**  No  Yes | 69.4 (10)  69.0 (10) | <0.001 |
| **Diabetes**  No  Yes | 68.9 (10)  73.8 (11) | <0.001 |
|  |  |  |
